# Evaluation of a decided sample size in machine learning applications

**DOI:** 10.1186/s12859-023-05156-9

**Published:** 2023-02-14

**Authors:** Daniyal Rajput, Wei-Jen Wang, Chun-Chuan Chen

**Affiliations:** 1 Institute of Cognitive Neuroscience, National Central University, Zhongda Rd, No. 300, Zhongli District, Taoyuan City, 320317 Taiwan, ROC; 2 Taiwan International Graduate Program in Interdisciplinary Neuroscience, National Central University and Academia Sinica, Taipei, Taiwan, ROC; 3 Department of Biomedical Sciences and Engineering, National Central University, Taoyuan, Taiwan, ROC; 4 Department of Computer Science and Information Engineering, National Central University, Taoyuan, Taiwan, ROC

**Keywords:** Sample size, Machine learning, Effect sizes, Criteria

## Abstract

**Background:**

An appropriate sample size is essential for obtaining a precise and reliable outcome of a study. In machine learning (ML), studies with inadequate samples suffer from overfitting of data and have a lower probability of producing true effects, while the increment in sample size increases the accuracy of prediction but may not cause a significant change after a certain sample size. Existing statistical approaches using standardized mean difference, effect size, and statistical power for determining sample size are potentially biased due to miscalculations or lack of experimental details. This study aims to design criteria for evaluating sample size in ML studies. We examined the average and grand effect sizes and the performance of five ML methods using simulated datasets and three real datasets to derive the criteria for sample size. We systematically increase the sample size, starting from 16, by randomly sampling and examine the impact of sample size on classifiers’ performance and both effect sizes. Tenfold cross-validation was used to quantify the accuracy.

**Results:**

The results demonstrate that the effect sizes and the classification accuracies increase while the variances in effect sizes shrink with the increment of samples when the datasets have a good discriminative power between two classes. By contrast, indeterminate datasets had poor effect sizes and classification accuracies, which did not improve by increasing sample size in both simulated and real datasets. A good dataset exhibited a significant difference in average and grand effect sizes. We derived two criteria based on the above findings to assess a decided sample size by combining the effect size and the ML accuracy. The sample size is considered suitable when it has appropriate effect sizes (≥ 0.5) and ML accuracy (≥ 80%). After an appropriate sample size, the increment in samples will not benefit as it will not significantly change the effect size and accuracy, thereby resulting in a good cost-benefit ratio.

**Conclusion:**

We believe that these practical criteria can be used as a reference for both the authors and editors to evaluate whether the selected sample size is adequate for a study.

**Supplementary Information:**

The online version contains supplementary material available at 10.1186/s12859-023-05156-9.

## Background

An appropriate sample size is a first and most crucial step in designing a faithful and ethical research study [1]. Generally, scientific studies can be divided into small (or under-sampled) and large studies. Small studies have a lower probability of producing true effects because of the higher chance of type I or II error [2, 3]. Specifically, Knudson and Lindsey [4] reported that type II errors of zero-order correlation and partial correlation increase from 7 to 21% and 29 to 85%, with sample sizes from 25 to 99. The results of small sample studies are particularly vulnerable to minor analytical manipulations that produce false-negative results [5, 6]. The winner’s curse reportedly inflates the effect size in small sample size studies because of random errors, selective analysis, selective outcomes reporting, and publication bias [7]. Thus, a sample size should be sufficiently large for producing scientific and statistical significance [8]. Scientists are advised to conduct large studies that can produce statistically true effects because of a higher statistical power. The outcomes of large studies are statistically more robust than small studies owing to less chance of inflated effect sizes and type I errors [9]. However, the question of adequate sample size remains to be solved. Indeed, it is commonly accepted that large sample sizes are not a substitute for good hypothesis testing [10]; Friston [10] suggested that a minimal sample size for producing statistically significant results is 16 subjects with a good effect size.

In machine learning, a few studies evaluated the impact of the sample sizes on accuracy [11]. For instance, Vabalas [12] investigated the impact of a range of simulated sub-datasets (20–1000) on the performance of support vector machine (SVM) and logistic regression (LR). They reported that small sample sizes resulted in higher accuracy (> 95%), whereas large sample sizes (100–1000) substantially decreased the accuracy between 60 and 70%. By contrast, Cui and Gong [13] found that the increment in sample size (ranging from 20 to 700) increased prediction accuracy using MRI data. In fact, Faber and Fonseca [14] demonstrated that increasing sample size beyond a range might not significantly improve results. Taken together, a small training sample size may exaggerate the accuracy of ML due to overfitting or random effects, whereas large-scale studies require more financial resources and consume more time [7, 12, 13]. Therefore, an adequate sample size is required for a reliable and efficient outcome of a study, but there is no practical guideline to evaluate the sample size, especially, under the conditions of ML performance.

In contrast, a sample size can be estimated statistically based on previous studies as a priori. For instance, the value of standard deviation, standardized mean difference, statistical power, and effect size based on previous studies can be used to determine the sample sizes [15–20]. However, these statistical approaches may measure inaccurate sample size due to inappropriate effect size calculation, the lack of experimental details, and publication bias [21]. An insufficient sample size has insignificant statistical power, which causes an adverse impact on the true effect and the reproducibility of the findings [22, 23]. Previous studies reported that effect size is used to calculate statistical power, such as a large effect size increases power while small effect size decrease power [24, 25]. Usually, scientists use Cohen’s equations to measure an effect size consisting of the mean and variance of two classes [26]. However, the mean and variance between two classes can be calculated in two ways: (1) average values of mean and variance and (2) grand values of mean and variance of data. Previous methods have not focused on the type of effect sizes (grand and average) and the difference between them in the sample size calculation and power analysis, which can adversely influence the outcomes of these measurements. Button [21] stated that a minor analytical manipulation could cause a substantial change in true effects, especially with small sample sizes. Thus, it is essential to accurately quantify the parameters for effect size, including the discrepancy between average and grand effect sizes, to measure an appropriate sample size.

In this study, we proposed that the effect sizes (average and grand) and the performance of ML together can be used to evaluate a sample size. Specifically, we examined the relationship between effect sizes (average and grand) and the performances of ML classifiers by using simulated datasets and three real datasets to derive two criteria for checking a selected sample size whether it is appropriate or not.

## Results

### Association between effect size and ML performance by employing simulated datasets

**Fig. 1:**
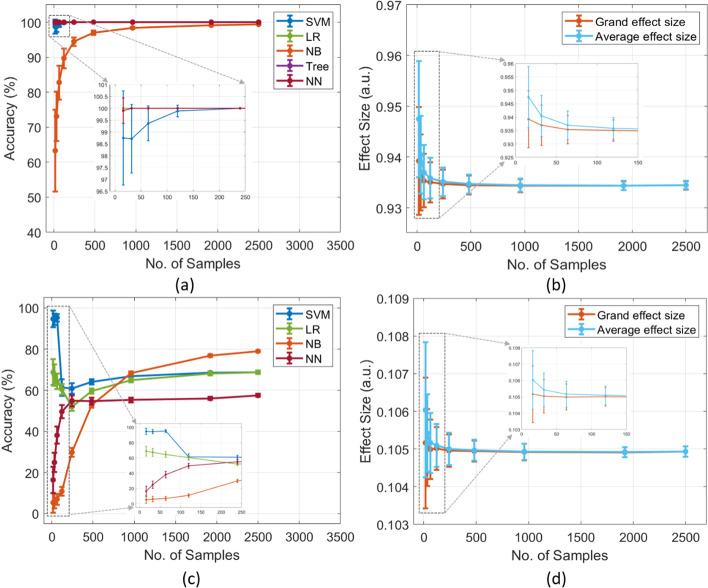
(**a**) ML performance of simulated datasets with (**b**) good effect sizes across the range of sample sizes,(**c**) ML performance of simulated datasets with (**d**) poor effect sizes across the range of sample sizes

We simulated the data by manipulating the effect size (good and poor) to examine their effects on the performance of classifiers with a range of sample sizes. Figure 1a and b illustrates that manipulation in effect size has a noticeable influence on the performance of classifiers. Most of the classifiers’ performance was more than 95%, when grand and average effect sizes were more than 0.9, except Naïve Bayes exhibited poor performance for small sample sizes. In addition, the variance in accuracy and both effect sizes were large in small sample sizes, which substantially decreased with increasing the sample sizes. In contrast, Fig. 1c and d demonstrates that ML performance was poor (less than 80%) when the grand and average effect sizes of the datasets were less than 0.2. In line with good datasets, the poor datasets also exhibited higher variance within sample sizes in ML accuracy (around 5% to 100%) and effect sizes (around 0.1034 to 0.1078) for small sample sizes.

### Effect of manipulation in data quality on ML performance and effect sizes

**Fig. 2:**
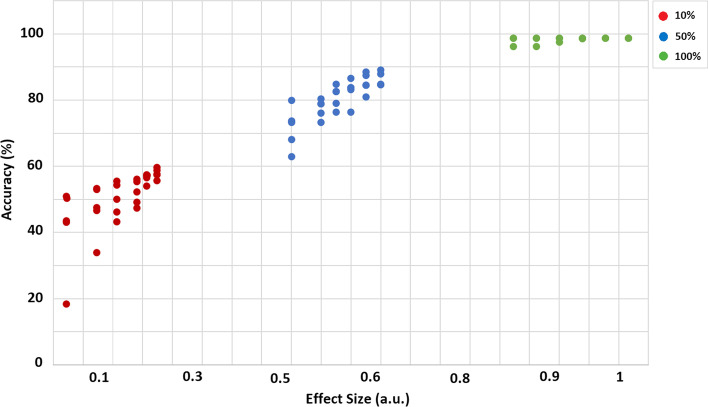
Performance of classifiers and effect sizes with different quality (10%, 50%, and 100%) of datasets. Note: a.u. is an arbitrary unit

We investigated ML performance and effect sizes by manipulating the quality of datasets (10%, 50%, and 100%). Figure 2 depicted that improving data quality significantly increased the ML performance from around 20 to 98% and effect size from about 0.1 to 0.9. The lower quality (10%) exhibited less than 70% performance, whereas increasing the quality from 50 to 100% substantially improved the accuracy by more than 70%. Besides, the datasets with 10% quality had smaller effect sizes around 0.2, while datasets with 50% and 100% quality had greater effect size around 0.55 and 0.9, respectively. Overall, the data quality of datasets exhibited a direct relationship with effect size and accuracy.

### The impact of sample sizes on classifier’s accuracy and effect size: two well-behaved arrhythmia and heart attack datasets

**Fig. 3:**
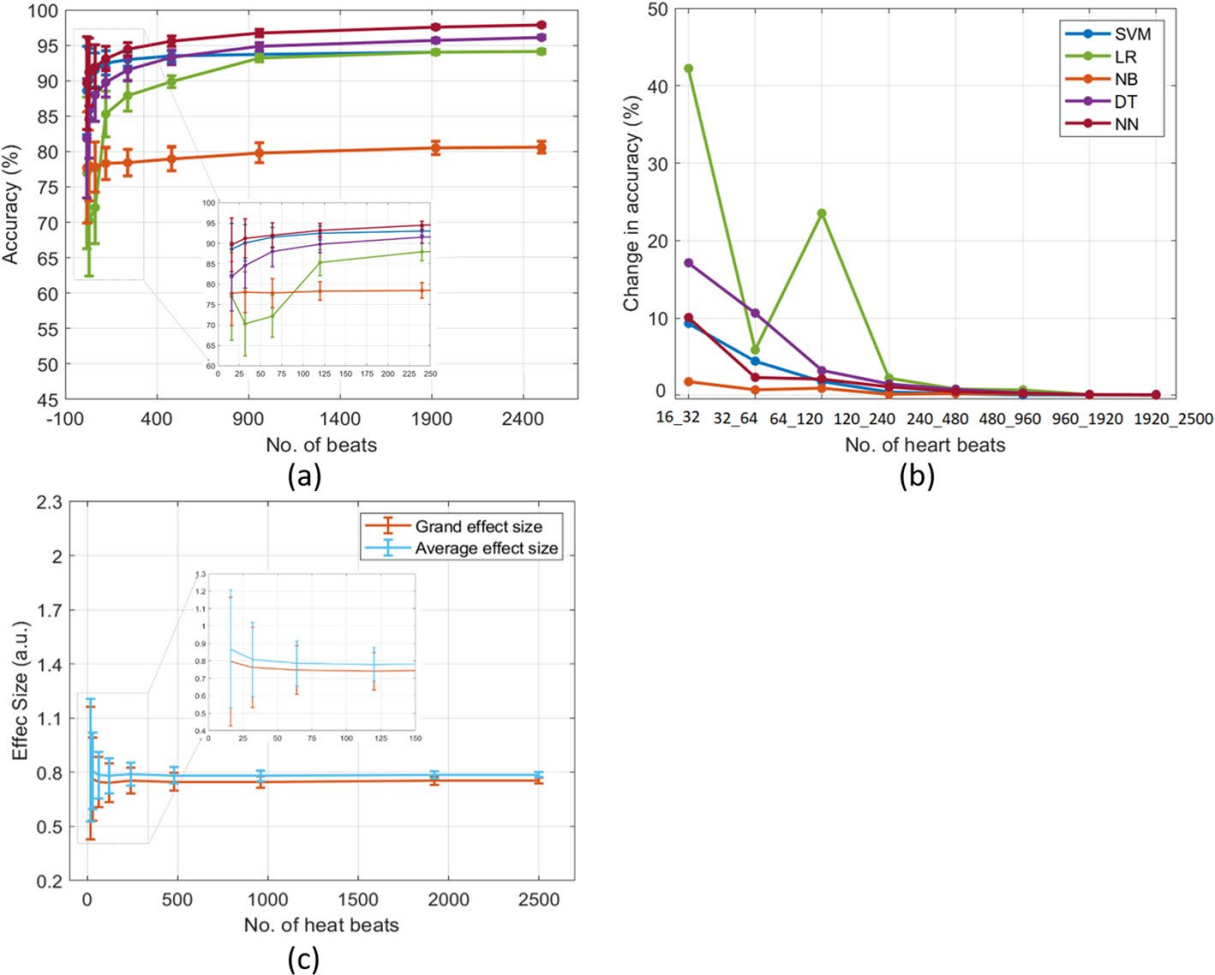
ML performance (**a**), changes in the performance between sample sizes (**b**), and average and grand effect sizes (**c**) of the arrhythmia dataset. Note: a.u. is an arbitrary unit

In order to understand the impact of different sample sizes (small to large) on the effect size and ML performance in real datasets, we used a large arrhythmia dataset that comprised 5000 samples. Figure 3 shows the ML performances with a 95% confidence interval (a), variation in accuracies between sample sizes (b), and the average and grand effect sizes (c). It can be seen that the classification accuracy increases with increasing the sample numbers, irrelevant to the classifiers (Fig. 3a). When the sample number is smaller than 120, all classifiers except NB exhibited greater variance in accuracy (between 68 and 98%), whereas increasing the sample sizes from 120 to 2500 reduced discrepancy in accuracy between 85 and 99% (Fig. 3a). Moreover, the accuracy of NN and SVM had more than 90% performance and outperformed with all sample sizes than other classifiers. LR exhibited significant variance in accuracy throughout different sample sizes, whereas NB was inefficient at separating the two classes. Considering the changes between sample sizes, the results revealed that samples less than 120 had greater relative changes in accuracy from 42 to 1.76% (Fig. 3b). On the contrary, samples greater than 120 showed relatively small changes in accuracy from 2.2 to 0.04% for all classifiers. Regarding the effect size, grand and average effect sizes were around 0.8, which indicated a good resolving power between the two classes (Fig. 3c). However, small sample sizes (specifically, 16, 32, and 64) depicted a higher variance in both effect sizes within a sample size, which shrank substantially with increasing the sample sizes.

**Fig. 4:**
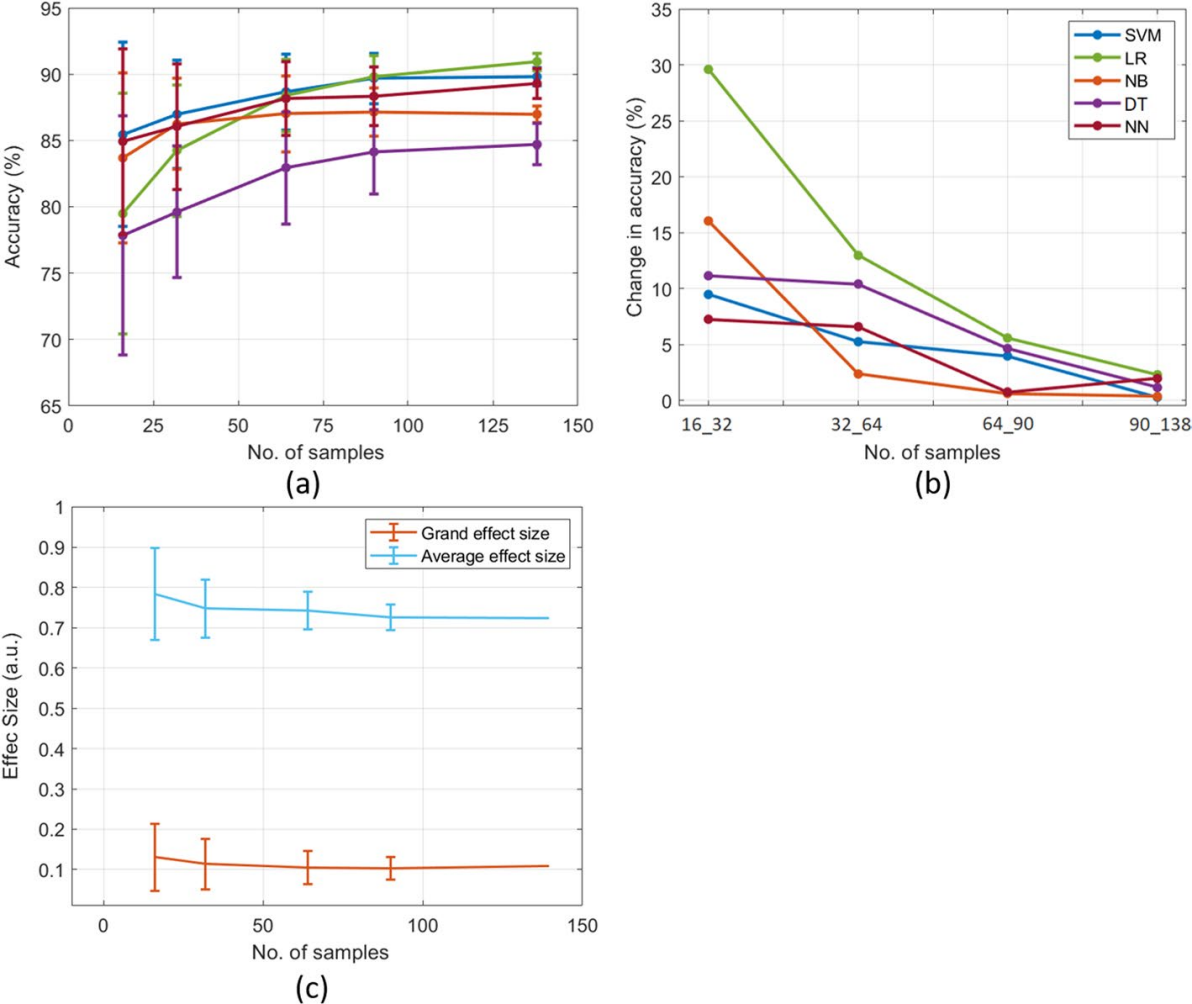
Classifiers’ performance (**a**), the discrepancy in the performance between sample sizes (**b**), and average and grand effect sizes (**c**) of the heart attack dataset. Note: a.u. is an arbitrary unit

Furthermore, Fig. 4a depicted that all classifiers had good performance (more than 80%) with heart attack datasets throughout the sample sizes, except DT and LR because their performances with small sample sizes (16 and 32) were around 78 to 79%. The performance of DT was poor (< 85%) compared to all classifiers throughout the different sample sizes. The variance within sample size was significantly higher in small sample sizes (between 67 and 93%), which reduced between 83 and 92% due to increasing sample sizes. Moreover, the changes in accuracy between small sample sizes (16–64) were 2.37% to 29.6% which gradually decreased between 5.57% and 0.37% after increasing samples from 60 to138 (Fig. 4b). In contrast to the arrhythmia dataset, the heart attack data showed a significant difference between grand and average effect sizes (Fig. 4c). The average effect sizes were between 0.7 and 0.8, whereas grand effect sizes were less than 0.2. Besides, 16 and 32 sample sizes showed significant variance in both effect sizes within the sample sizes, which noticeably decreased with the increase of sample sizes.

Both datasets exhibited that increasing sample sizes improved ML performance (> 80%) and reduced the discrepancy among the different classifiers. The average and grand effect sizes were more than 0.5 in the arrhythmia dataset, whereas only the average effect size was more than 0.5 in the heart attack dataset.

### The impact of sample sizes on classifier’s accuracy and effect size: an indeterminate sleep dataset

**Fig. 5:**
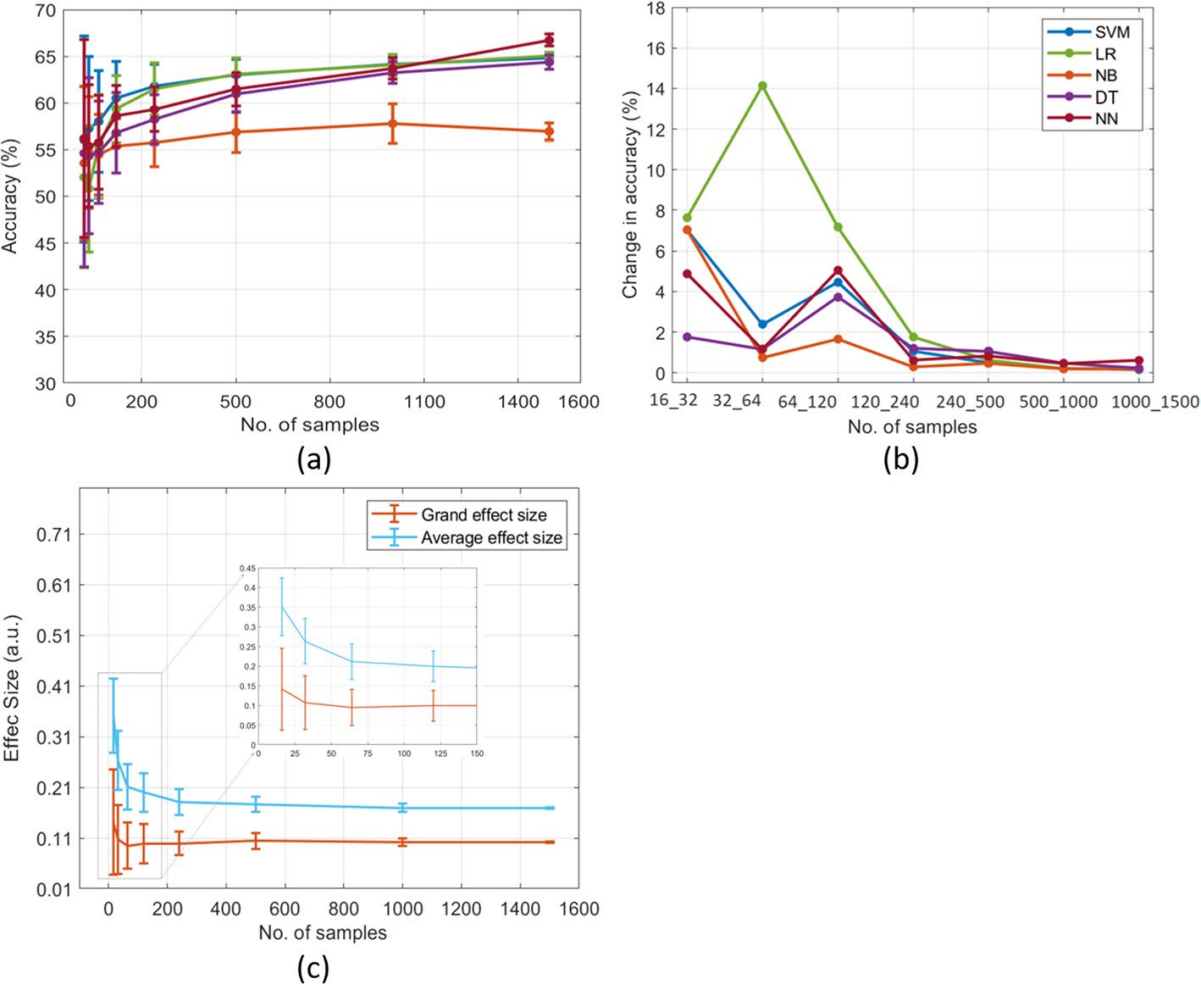
Manipulation in the sample sizes of the indeterminate dataset to assess effect size (**a**) and ML performance (**b**) with the rate of change of the accuracy (**c**) between sample sizes. Note: a.u. is an arbitrary unit

We used the sleep dataset with indeterminate properties to investigate the sample size effects. Figure 5 shows the ML performance with a 95% confidence interval (a), the rate of change of accuracies between the sample sizes (b), and the sample size-dependent average and grand effect sizes (c). The ML results showed that the sleep dataset with small sample sizes (16–120) had performance between 51 and 60%, whereas increasing sample sizes to more than 120 improved the performance from around 60 to 67%. NB exhibited the worst performance between 51 and 57% across all sample sizes (Fig. 5a). The accuracy changes between small sample sizes (16–120) were 0.73 to 14.14%, which gradually decreased from 7 to 0.17% with the increase of samples (120–1500, Fig. 5b). Overall performance of the sleep dataset was poor (less than 70%) throughout the sample sizes, which concluded that all classifiers were unable to separate the two classes. Apart from ML performance, the mean average and grand effect sizes were reduced with increasing sample sizes from 0.35 to 0.168 and 0.14 to 0.1, and their variances in the effect sizes from 0.43 to 0.16 and 0.248 to 0.035, respectively (Fig. 5c). Altogether, the sleep dataset had less than 0.5 average and grand effect sizes, which is considered a poor effect size according to Cohen’s scale.

## Discussion

This study examined the impact of sample numbers on the effect size and ML classifiers’ performance to design criteria for evaluating an appropriate sample size by using simulated datasets and three real datasets with different data properties. The simulated results demonstrate that a dataset with good effect sizes improves ML performance compared to a dataset with poor effect size. Besides, data quality significantly improves the performance and effect size throughout the sample sizes, especially small sample sizes. On the other hand, the arrhythmia and heart attack datasets elicit that a well-behaved dataset has good discriminative power between two classes due to good effect sizes and higher classification accuracy, while the variances in both effect sizes and ML performance shrink with the increment of samples. Importantly, the difference in the average and grand effect sizes was significantly higher in the heart attack dataset compared to other datasets. By contrast, for indeterminate datasets, both effect sizes and classification accuracies were poor throughout the sample sizes. Due to the nature of random sampling, the effect sizes vary across the same sample size. Especially, in the small sample size of 16, the effect sizes and ML accuracies dramatically changed in all datasets. Nevertheless, the accuracies reach a plateau after a critical sample size with a good effect size. Based on the above findings, we derived two criteria for evaluating a sample size by combining the conventional statistics of effect sizes (average and grand) and the accuracy of machine learning approaches.

### Relationship between effect sizes and ML performance under different data quality by using simulated datasets

We examined how good and poor effect sizes (both average and grand) affect the performance of different classifiers. The results elicited that the good effect sizes (more than 0.8) exhibited higher performance (> 90%), while poor effect sizes (less than 0.2) showed poor ML performance (< 80%). Previous studies reported that a good effect size demonstrates a higher difference between two groups, whereas a small effect size had a trivial difference [27]. Similarly, machine learning techniques assessed multiple variables to differentiate two or more groups, such as patients from healthy controls or one disease from others [28, 29]. Hence, ML classifiers easily differentiate the two groups when the effect size is large. Besides, our results demonstrated that quality of data significantly improved the effect size and ML performance. Previous studies reported that small sample size studies are inappropriate for research studies because of low statistical power, inflated true effect, and minor analytical manipulations [21]. In this study, we manipulated the quality of one of the poor classes by replacing its 10%, 30%, and 50% data with good data. Our simulated results elicited that poor data quality (between 10 and 30%) exhibited inflated poor effect sizes (from around 0.1 to 0.3) and poor ML performance (from about 20% to 70%), especially with small sample sizes. However, increasing the data quality (50% and 100%) exhibited significant improvement in effect size (> 0.5) and ML accuracy (> 80%). Importantly, small sample sizes with 100% quality showed good effect sizes (around 0.9) and the performance (around 95%), and changes between sample sizes were minute across all sample sizes (Figs. 1 and 2). Thus, our simulated results conclude that increasing the data quality substantially improves effect sizes and accuracy from small to large sample sizes. Besides, a small sample size can be appropriate for a study if it has good quality because it will not be affected by winner curse and minor analytical manipulations.

### The difference in average and grand effect size calculations and its impacts on sample size calculation

Previous studies used effect size in power analysis and designed a few formulae for sample size calculation [24]. However, previous studies reported that the effect size calculation in these formulae was biased due to improper calculation. For example, a review study reported that effect size could be inflated due to small sample sizes, publication bias, improper study design, biased selection of parameter values for effect size calculation (such as mean and variance from different studies), and lack of experimental information [21]. Previous research mainly focused on the aforementioned limitations of effect size, although no study reported the effect of the difference between average and grand effect sizes on sample size calculation. This is the first study that has examined the discrepancy between average and grand effect sizes to calculate a suitable sample size. This study found that the simulated and heart attack datasets exhibited a significant difference between average and grand effect sizes compared to other datasets. Our results also revealed that one of the effect sizes should be greater than 0.5 to attain good ML accuracy. Therefore, it is essential that average and grand effect sizes of a dataset should be measured for an accurate calculation of sample size by avoiding any bias due to dissimilarity between them.

Furthermore, the values for the calculation of effect size in existing methods are taken from previous studies, which surely carry a few potential biases such as publication bias, improper study design, biased selection of parameter values (mean and variance), and lack of experimental [21]. Importantly, a minor analytical manipulation in calculation could cause a substantial change in true effects [21]. The proposed criteria for effect size estimation use current data, which may help to avoid biases that can occur when calculating effect size based on previous studies. It is important to note that the effect size calculation based on current data can help to mediate biases introduced by differences in study design and parameter selection between previous studies. However, if a study uses current data with inadequate design or parameter selection, it may negatively impact the effect size, as seen in the sleep datasets in this study which showed poor effect size and accuracy. Nonetheless, the criteria can be used to address this issue by evaluating the effect sizes after taking a few steps such as increasing sample size, adding parameters, and/or revising study design.

### Sample size evaluation and two criteria

The frequently used methods for sample size determination are using the effect size or standardized mean difference [15, 30]. However, their effect size calculations are biased due to the improper selection of parameter values, such as the selection of mean from prior studies. In addition, average and grand effect sizes showed a significant difference in this study, which was ignored in previous approaches, and this difference can adversely affect the sample size calculation. Regarding ML, there is a scarcity of ML research for sample size determination [11]. A review study reported that a few methods, such as curve-fitting, cross-validation, and linear discriminant analysis, have been used to investigate sample size. Yet, the results are rather inconsistent because of some potential limitations, such as bias-variance tradeoffs, over-sampling, algorithms, and feature size [11, 31, 32]. In addition, a few methods are available to select data for training classifiers, such as sub-modular selection [33–35]. In theory, reducing the samples leads to a greater variance [21]. However, if the removed samples originally contributed to large data variance (for example, close to outlier), this may result in a smaller variance. Therefore, it is very difficult to establish one robust relation between sample selection and effect size. Given that the effect size is data-dependent, methods like the sub-modular selection can serve as a pre-process procedure before checking the sample size using the proposed criteria.

Furthermore, we investigated simulated (good) and two publically available datasets in this study: arrhythmia and heart attack. Several studies have used these datasets to develop efficient neural network and ML models for predicting cardiovascular diseases [37–40]. For instance, Kim et al. [37] developed a novel arrhythmia classification algorithm using the MIT-BIH Arrhythmia dataset, achieving an average sensitivity of 98.00%, specificity of 97.95%, and accuracy of 98.72% in classifying six types of heart beats. Another study used the heart attack dataset to identify key features related to heart attacks with machine learning models, achieving accuracies between 81.97 and 90.16% [39]. We used these datasets in our current study because they have strong discriminative power. We analyzed the effect sizes and machine learning performance of these datasets across different sample sizes, unlike previous studies which only used machine learning on the entire sample size. We found that the accuracies for both simulated (good) and real datasets were more than 80% owing to high average and grand effect sizes (≥ 0.5), except grand average of heart attack (0.2 ≤ , see Figs. 3 and 4). This result confirmed the theoretical conclusion that an effect size of more than 0.5 exhibited a higher accuracy of more than 80% [10], irrelevant to the sample sizes. However, when data have effect sizes smaller than 0.5, it is essential to have adequate samples or features as the effect size and classification accuracy increase with the increment of samples. Moreover, we suggested that the difference between sample sizes should be less than 10% because the rate of change in accuracy decreased owing to increasing sample sizes (see Figs. 3b, 4b, and 5b). The results revealed that, in all datasets, small sample sizes showed a greater change in ML performance which reduced to a minute level (such as around 0.04%) by increasing sample sizes. In contrast, the simulated (poor) and indeterminate dataset exhibited poor effect sizes of less than 0.2, whereas the ML accuracy of all classifiers was less than 80%. Overall, the results revealed that the increase in sample sizes significantly improved the performance and reduced standard errors [36]. Notably, the performance of classifiers and effect size were stable after a critical sample size, despite the different classifiers may have different critical sample sizes. In addition to accuracy, we also examined other metrics such as AUC-ROC, precision, recall, and F1 Score to evaluate a classifier (e.g. logistic regression) and their results showed comparable performance (see Additional file 1: Supplementary Figure 1). After assessing our analytic results, we proposed two criteria for evaluating the study sample size.

*Criteria 1:* Calculate average and grand effect sizes of data. One of the effect sizes of a decided sample size should be equal to or more than 0.5 according to Cohen’s scale [19].

*Criteria 2:* ML accuracy of a decided sample size should be equal to or more than 80%. When multiple sample sizes are compared, the change of accuracies should be smaller than 10% with the increase of samples beyond a desired accuracy (e.g., accuracy ≥ 80%).

In this way, we can expect that the increment in samples after this practical sample size will not produce a beneficial effect as it will not significantly change the effect size and ML performance. The effect size alone is not robust for evaluating the sample size when the sample size is small. This is because the random sampling effect could result in large variability of effect sizes for a given sample size. Moreover, it should also be noted that sleep datasets in this study exhibited poor ML performance and effect size. Hence, there is a possibility that a dataset may not comply with two criteria because of three possible situations and these conditions can be fixed by taking appropriate response: (1) if the accuracy, but not the effect size, increases with the increment of samples, more samples and different features are suggested.; (2) if the increase of samples can’t improve either accuracy or effect size, this is mainly because the features are not representative or informative. In situations like this, we would suggest that one may modify the experimental design and acquire proper features; (3) if the effect size increases with the increment of samples but not passes the criteria, more samples are required. This is because an improvement of effect size will surely lead to a better accuracy.

### Questions about generalization of this guideline and the best classifiers

Biomedicine comprises divergent data types, for instance, imaging informatics, bioinformatics, clinical informatics, and public health informatics [41]. This makes it a big challenge to generalize the results of a special datatype-classifier by comparing with others. By contrast, the effect size is a universal and intrinsic statistical property, irrelevant to data types [42–45]. We, therefore, used three datasets with different statistical effect sizes (i.e. good and indeterminate) to make possible the comparison between different datatype-classifier pairs. Despite it is not possible to generalize our guideline to all biomedical applications, we believe these two criteria can be implemented in most settings.

Regarding the question of selecting the best classifier, it is well accepted that different classifiers have specific evaluation procedures [46] and may best suit different data types [47]. Therefore, the naive bayes requires more sample to train compared to the neural network in this work may be because of the data used. Similar results that Naive Bayes classifier outperformed the neural network classifier have been reported in different applications [48] (see [49] for a review). Additionally, sample size significantly influences the performance of classifiers, as seen in our results and in a review study that the large sample sizes depict relatively precise and similar accuracies among classifiers [47]. Taken together, both simple size and datatype affect the performance of classifiers. Therefore, there is no guideline for selecting a best classifier but only “trial and error”. Nevertheless, we reported the impacts of sample sizes on five frequently used ML classifiers as examples and proposed to consider the effect sizes for evaluating the sample size. Our results did show consistent patterns of accuracy across different classifiers. Further study may explore this best classifier issue to provide the guideline for selecting ML methods.

## Conclusion

This study examined the impact of sample numbers on the effect sizes (average and grand) and the ML classifiers’ performance. We observed that effect sizes and the ML accuracies reach a plateau after certain samples. A small sample can be sufficient for a study when a dataset has high-quality data. Importantly, the discrepancy between average and grand effect size should be considered when calculating sample size or power analysis. Based on the above findings, we derived two criteria for evaluating a sample size by combining the conventional statistics of effect sizes (average and grand) and the accuracies of machine learning approaches. We believe that these criteria can be used as a reference to determine whether a selected sample size is adequate for both the authors and editors who handle these types of studies.

## Methods

### Simulated data

We used Eq. 1 to generate simulated datasets to examine the relationship between the effect sizes (average and grand) and the performance of ML classifiers by manipulating effect sizes of different sample sizes (ranging from 16 to 2500) with 100 variables. Here, we manipulated the effect sizes of datasets by changing the mean and variance values in Eq. 1. Here, we chose 16 samples as the smallest sample size because previous studies considered 16 to 32 samples as a small sample size [10, 50]. First, the impact of poor and good effect sizes on ML classifiers’ accuracies was investigated by generating two kinds of datasets based on Cohen’s scale: poor datasets (effect sizes between 0.01 and 0.2) and good datasets (effect sizes between 0.5 and 1.4) [26]. Second, we used the effect size as an indirect index of dataset quality. We manipulated the variance of certain portion of all features across a range of sample sizes (between 16 and 2500) and examined their effect on ML performance. Thus, the data were first generated with similar variance of all features between groups. We introduced a perturbation into the feature variance of one of the groups by substituting a certain portion (10%, 30%, or 50%) of features in the bad datasets with same amount features from good datasets (effect size > 0.5). This procedure can alter the variances of the substituted features and lead to a better grand effect sizes, indirectly reflecting the dataset quality. Because 10%, 30% and 50% are the ratio of total features in a sample, they are irrelevant to the sample size.

1 $${\text{Ds}} = \mu \times \sigma + \varepsilon$$

here, Ds are the simulated data, μ and σ represent mean and variance of data, whereas ε denotes the random noise.

### Real data preparation

In order to understand the relationship between effect size and ML performance from small to large sample sizes, we further extended the investigation from simulated datasets to real datasets. We used three datasets, arrhythmia, heart attack, and sleep datasets, to derive criteria for determining an appropriate sample size. The first dataset, the heart attack data, was downloaded from the Cleveland dataset of the UCI Machine Learning Repository (available at http://archive.ics.uci.edu/ml/datasets/Heart+Disease). This dataset, comprising 303 patients (206 men and 97 women with mean age of 54 years) and 76 attributes, was recorded between May 1981 and September 1984. Because the patients are disproportionately distributed in two classes: less chance of heart attack (138 patients) and more chance of heart attack (165 patients), we chose only 276 subjects’ data to balance the samples in each class (138 samples). Furthermore, it has been shown that 14 out of the 76 attributes are sufficient for appropriate classification results. Hence, we chose 14 attributes in this study.

The second dataset, arrhythmia data, was the MIT-BIH Arrhythmia dataset from PhysioNet (https://physionet.org/content/mitdb/). The dataset was developed to evaluate and design algorithms for detecting arrhythmia by the Beth Israel Hospital Arrhythmia Laboratory between 1975 and 1979 [51, 52]. It contains 48 half-hour recordings of two-channel ECG (sampling rate = 360 Hz, bandpass filtered from 0.1 to 100 Hz). The dataset had 109,000 beats and 188 attributes which provide information of ventricular and supraventricular arrhythmias, conduction abnormalities, pacemaker rhythms, and artifacts. At least two cardiologists manually reviewed all these beats. The dataset was divided into five categories to evaluate the efficacy of arrhythmia identification algorithms: normal (N), supraventricular (S), ventricular (V), fusion (F), and indeterminate (Q) heartbeats [53]. In this study, we used two classes of heartbeats (normal and indeterminate heartbeats), and each category consisted of 2500 heartbeats. Because of missing values in the dataset, we used only the 100 attributes.

The third dataset was a sleep dataset that contained information about the factors affecting the sleep cycle during menstruation, such as sleep quality, waking time, sleep latency, headache, swollen tummy, and concentration. The dataset comprised 120 sleep diaries, and each diary represented one woman’s sleep cycle data over a period of one month. The dataset was divided into two categories: women with abnormal and normal sleeping cycles. Overall, the dataset consisted of 3360 samples (120 diaries × 28 days) with 57 continuous and categorical variables. However, we used 3000 samples (1500 in each category) after removing missing values.

All datasets were randomly partitioned into subsets with different samples, starting from 16 toward the whole datasets. With different sample numbers, we measured the effect sizes and ML performances. We repeated this procedure 100 times to mimic the randomized sampling process. We chose 80% of accuracy as a threshold based on previous sample size calculation method, namely power analysis. In power analysis, power is the probability of rejecting a false null hypothesis and a sufficient sample size should maintain to obtain a power as high as 80% or more [54].

### Calculation of average and grand effect sizes

Cohen’s formula [26] was employed to calculate the effect size of the given samples as follows:

2 $$d = \frac{{\overline{{x_{1} }} - \overline{{x_{2} }} }}{{S_{pooled} }}$$

3 $$S_{pooled} = \sqrt {\frac{{\left( {n_{1} - 1} \right)sd_{1}^{2} + \left( {n_{2} - 1} \right)sd_{2}^{2} }}{{ss_{1} + ss_{2} - 2}}}$$

#### Average effect size

Average mean and standard deviation across samples

4 $$\mu_{avg} = \frac{\sum x}{{ss}}$$

5 $$\sigma_{avg} = \sqrt {\frac{{\sum {\left( {x - \frac{{\overline{x}}}{ss}} \right) ^{2} } }}{ss - 1}}$$

Pooled standard deviation (PSD) based on average variance

6 $$S_{{Pooled_{avg} }} = \sqrt {\frac{{\mathop \sum \nolimits_{i} \left( {ss_{{1_{i} }} - 1} \right) \times \left( {\sigma_{{avg_{1} }} } \right)^{2} + \mathop \sum \nolimits_{i} \left( {ss_{{2_{i} }} - 1} \right) \times \left( {\sigma_{{avg_{2} }} } \right)^{2} }}{{\left( {ss_{{1_{i} }} + ss_{{2_{i} }} } \right) - 2}}}$$

Measurement of Cohen d by applying average pooled standard deviation and mean

7 $$d_{avg} = \frac{{\mu_{{avg_{1} }} - \mu_{{avg_{2} }} }}{{\sqrt {\frac{{\mathop \sum \nolimits_{i} \left( {ss_{{1_{i} }} - 1} \right) \times \left( {\sigma_{{avg_{1} }} } \right)^{2} + \mathop \sum \nolimits_{i} \left( {ss_{{2_{i} }} - 1} \right) \times \left( {\sigma_{{avg_{2} }} } \right)^{2} }}{{\left( {ss_{{1_{i} }} + ss_{{2_{i} }} } \right) - 2}}} }}$$

Average effect size across each sample size

8 $$\overline{d} = \frac{{\mathop \sum \nolimits_{j} d_{{avg_{j} }} }}{vs}$$

#### Grand effect size

Calculation of grand mean and standard deviation

9 $$\mu_{g} =\frac{\sum_{j}[\frac{\sum{x}}{ss}]}{vs}$$

10 $$\sigma_{g} = \frac{{\mathop \sum \nolimits_{j} \left[ {\sqrt {\frac{{\sum \left( {x - \frac{{\overline{x}}}{ss}} \right)^{2} }}{ss - 1}} } \right]}}{vs}$$

Pooled SD by employing grand mean and variance

11 $$S_{Pooled\_g} = \sqrt {\frac{{\mathop \sum \nolimits_{i} \left( {ss_{{1_{i} }} - 1} \right) \times \left( {\sigma_{{g_{1} }} } \right)^{2} + \mathop \sum \nolimits_{i} \left( {ss_{{2_{i} }} - 1} \right) \times \left( {\sigma_{{g_{2} }} } \right)^{2} }}{{ss_{{1_{i} }} + ss_{{2_{i} }} - 2}}}$$

Compute Cohen d with grand values of the parameters

12 $$d_{g} = \frac{{\mu_{{g_{1} }} - \mu_{{g_{2} }} }}{{\sqrt {\frac{{\mathop \sum \nolimits_{i} \left( {ss_{{1_{i} }} - 1} \right) \times \left( {\sigma_{{g_{1} }} } \right)^{2} + \mathop \sum \nolimits_{i} \left( {ss_{{2_{i} }} - 1} \right) \times \left( {\sigma_{{g_{2} }} } \right)^{2} }}{{ss_{{1_{i} }} + ss_{{2_{i} }} - 2}}} }} = \frac{{\mu_{{g_{1} }} - \mu_{{g_{2} }} }}{{S_{Pooled\_g} }}$$

here, *d* is Cohen’s *d* effect size, $S_{pooled}$ is the pooled standard deviation, sd_1_ and *s*d_2_ are the class-specific standard deviation,$x_{1} \;{\text{and}}\;x_{2} { }$ are the values of class 1 and 2, *i* and *j* both are used as the index of samples and variables, *vs* are variable size and ss_1_ and ss_2_ are the class-specific numbers of samples, respectively. Besides, $avg$, and $g$ terms are used to describe average and grand values of the mean and variance. Usually, in the Cohen d’s scale, the effect size of 0.2 represents a trivial difference, while an effect size of 0.5 or more considers a significant difference [19]. In order to get an immaculate Cohen's d, $\overline{{x_{1} }} ,\overline{{x_{2} }}$, *s*_1_ and *s*_2_ must be deterministic; however, they are usually taken from previous studies [16, 19, 55]. In addition, mean and variance also show a significant discrepancy in the calculation of grand and average effect sizes. Therefore, the derived sample size is biased toward the referenced studies because of the selection of effect size (grand or average), different or incomplete detail of experimental design, publication bias, or small effect sizes [24]. Instead of using previous studies, this study retrogradely calculated the effect sizes with respect to the given data and also calculated grand and average effect sizes.

Regarding the ML algorithms, we compared five frequently used supervised ML methods—SVM, LR, decision tree (DT), neural network (NN), and Naïve Bayes (NB), to examine the effect of different sample sizes (small to large) on ML performance. We employed ten-fold cross-validation to quantify the accuracy.

## Supplementary Information

**Additional file 1**. **Supplementary Figure 1:** Assessment of logistic regression classifier with different methods by using (a) arrhythmia dataset (b) sleep dataset.

## Data Availability

The MIT-BIH Arrhythmia dataset analysed during the current study are available in the PhysioNet repository, https://physionet.org/content/mitdb/. The heart attack dataset analysed during the current study are available in the UCI Machine Learning Repository, http://archive.ics.uci.edu/ml/datasets/Heart+Disease. The sleep dataset used during the current study are available from the corresponding author on reasonable request.
