# Supplementary material for: Evaluation of a decided sample size in machine learning applications
Source: BMC Bioinformatics. 2023 Feb 14;24:48. doi: 10.1186/s12859-023-05156-9 (PMC9926644; doi:10.1186/s12859-023-05156-9)
Supplement: Supplementary file 1 — Additional file 1. Supplementary Figure 1: Assessment of logistic regression classifier with different methods by using (a) arrhythmia dataset (b) sleep dataset. [file 12859_2023_5156_MOESM1_ESM.docx]

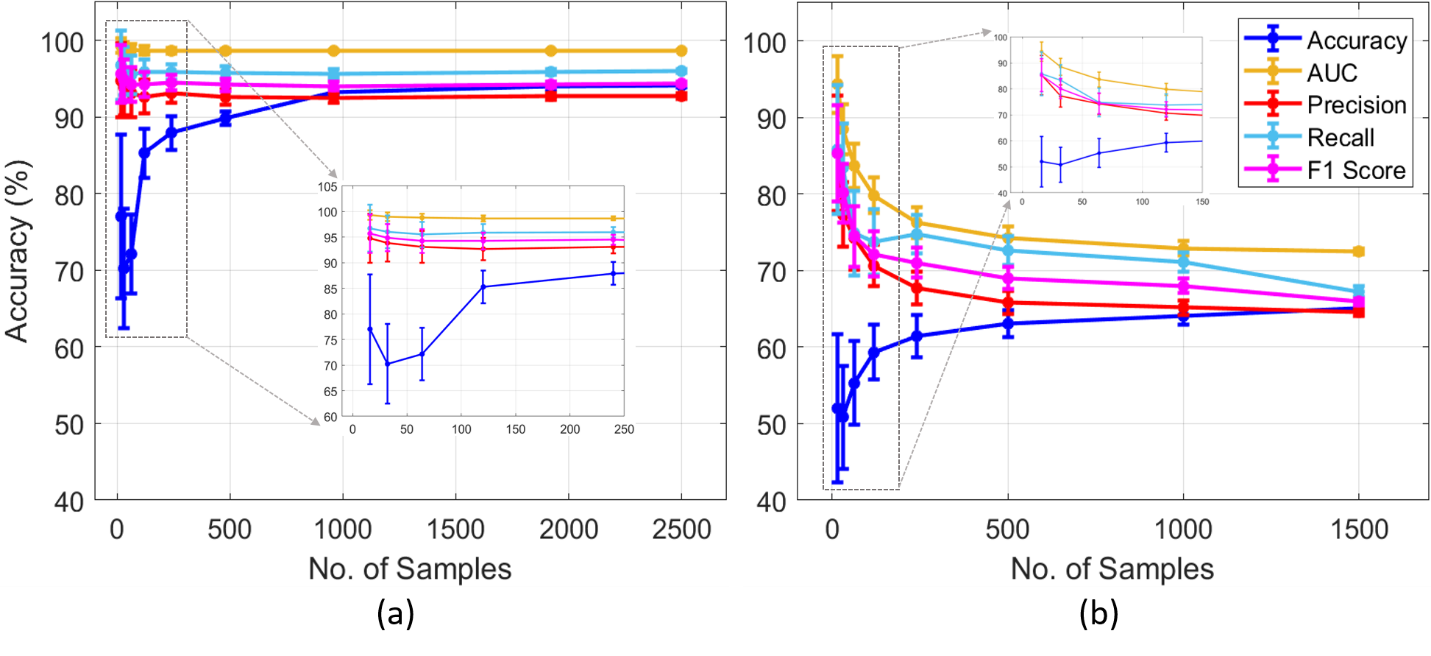


Supplementary Figure 1: Assessment of logistic regression classifier with different methods by using (a) arrhythmia dataset (b) sleep dataset.
